# Temporal activity and detection rates of chilla (*Lycalopex griseus*) in Tierra del Fuego, Argentina

**DOI:** 10.1002/ece3.11586

**Published:** 2024-06-14

**Authors:** Maximilian L. Allen, Andrew T. L. Allan

**Affiliations:** ^1^ Illinois Natural History Survey, Prairie Research Institute University of Illinois Champaign Illinois USA; ^2^ Department of Anthropology Durham University Durham UK

**Keywords:** chilla, grey fox, invasive species, nocturnal, temporal activity, Tierra del Fuego

## Abstract

Designing mitigation strategies for invasive species requires a clear understanding of their ecology and behaviour. Chilla (or grey fox; *Lycalopex griseus*) were introduced to Isla Grande de Tierra del Fuego (Tierra del Fuego Island) in 1951 to control European rabbit (*Oryctolagus cuniculus*) populations. Although this management strategy was unsuccessful, the chilla spread across the island and are now considered invasive. Despite this, there is a lack of research concerning their ecology and behavioural patterns, particularly on the Argentinian side of the island. We assessed the detection rates and temporal activity patterns of chillas using camera traps in the Argentinian region of Tierra del Fuego Island. Chilla had average detection rates of 61.7 (SD ± 33.3, range = 13.5–105.7) per 100 trap nights. Although analysis by clock time suggested cathemeral activity patterns, when analysed by sun time the chillas exhibited distinct nocturnal activity patterns. These findings offer the first information on the detection rates of chilla on the Argentinian side of Tierra del Fuego Island and reveal new insights into their temporal activity patterns, providing an important basis for future research that may aid the development of more effective management and conservation strategies.

## Graphical abstract

The temporal activity of chillas in Tierra del Fuego by (a) clock time and (b) sun time.

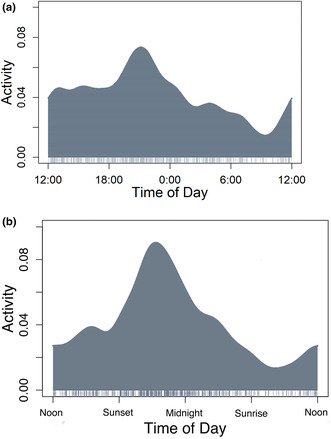

## 1 INTRODUCTION

Invasive species are a key component of global change, and understanding the ecology and behaviour of these species is important for effective management and conservation efforts aimed at mitigating their impacts on natural ecosystems. Invasive species are characterized by their ability to rapidly reproduce and spread into new environments, posing substantial threats to biodiversity and ecosystem functions (Simberloff et al., 2013). Studying the ecological and behavioural traits of invasive species can provide insights into the underlying mechanisms driving their expansion and therefore provide information to help predict their ecological consequences and apply evidence‐based management actions (Mack et al., 2000).

Chilla (*Lycalopex griseus*, also known as Argentine grey foxes) represent an interesting case as an understudied mesocarnivore across their range that is considered to be an invasive species in Tierra del Fuego. Chilla are considered of Least Concern by the International Union for Conservation of Nature (IUCN; Lucherini, 2016) and have received relatively little attention in the context of global carnivore research, as is typical of species endemic to the Southern Hemisphere (Allen et al., 2019; Nuñez et al., 2019). A total of 24 chillas were introduced to Tierra del Fuego Island (Isla Grande de Tierra del Fuego) in 1951 to control invasive European rabbits (*Oryctolagus cuniculus*) (Zurita et al., 2023). Tierra del Fuego Island is the largest island in South America at approximately 48,000 km^2^ (Zurita et al., 2023) and is split between Chile and Argentina. Spreading at rate of at least 7.7 km per year across the island (Jaksić et al., 2002), the foxes thrived in this novel environment, with ~22,000 individuals estimated to be spread across the Chilean side of the island in 2021 (Zurita et al., 2023). Despite this estimate falling to around 13,000 in 2022, largely due to the increasing population of free‐living dogs and hunting pressure (Zurita et al., 2024), chillas have unquestionably become a major part of the natural ecosystems and anthropogenic landscapes; however, abundance data from the Argentinian side of the island have so far been lacking (Zurita et al., 2023). With no landforms dividing the island, chillas in Chile and Argentina are considered part of the same population; however, Chile has a hunting quota of 10 specimens per hunter per day (Zurita et al., 2024), whereas chillas in Argentina have zero protection (Zurita et al., 2023). Thus, although chillas should have access to similar habitats and resources across both sides of the island, they may be under differing levels of risk from humans and free‐living dogs.

Beside their status as an invasive species, there remains a notable gap in our understanding of the ecology and population dynamics of chillas on Tierra del Fuego Island (Zurita et al., 2023) and throughout their range (Lucherini, 2016; Muñoz‐Pedreros et al., 2018). Although lagomorphs often make up a substantial part of the diet of chillas (Muñoz‐Pedreros et al., 2018; Zúñiga et al., 2022), lagomorphs are now relatively rare in their diet on Tierra del Fuego Island due to the dramatic decline in European rabbit populations since the introduction of the myxoma virus (Zurita et al., 2023). On the Chilean side of the island, chilla were found to feed on native rodents, birds, reptiles and invertebrates, with ~40% of individuals also feeding on carrion from domestic livestock, mainly *Ovis aries* (Jaksić & Yáñez, 1983). In particular, chilla are suggested to have played a major role in the decline of ruddy‐headed geese (*Chloephaga rubidiceps*) (Madsen et al., 2003) and native bird populations generally, leading them to be considered an invasive species (Zurita et al., 2023, 2024). Chilla may have a competitive relationship with the larger sympatric culpeo fox (*L. culpaeus*) on Tiero del Fuego Island (Jaksić et al., 2002). Although no other dominant native wildlife exists on the island, the most recent population estimate indicated a decline of >50% in chilla abundance in the previous 15 years, likely driven by direct killing by free‐living dogs (Zurita et al., 2024). On the mainland of southern Chile, free‐living dogs seem to influence chilla space use (Silva‐Rodríguez et al., 2010), suggesting the chillas of Tierra del Fuego Island may adopt similar spatiotemporal avoidance strategies. Research from the Chilean side of the island suggests chillas exhibit a crepuscular temporal activity pattern (Zurita et al., 2024), which may be important for chillas to avoid persecution/attacks from dogs and humans, but no research has investigated activity patterns in the Argentinian side of the island.

We used cameras placed by the Wildlife Conservation Society on the Argentinian side of Tierra del Fuego Island to assess the detection rates and temporal patterns of chillas, to better understand their ecology and behaviour. We first calculated the detection rates of chillas as a relative measure of abundance for reference in future studies. We then assessed their temporal activity by clock time and sun time (to account for each day having different lengths). Given that day length can be as much as 10 hours longer in the summer than the winter on Tierra del Fuego, we hypothesized that there would be significant differences in chilla activity patterns when analysed by clock time versus sun time. We then predicted that their activity would peak during nocturnal hours as this should minimize the likelihood of chillas encountering humans and free‐living dogs, their principal threats on the island.

## 2 MATERIALS AND METHODS

**FIGURE 1:**
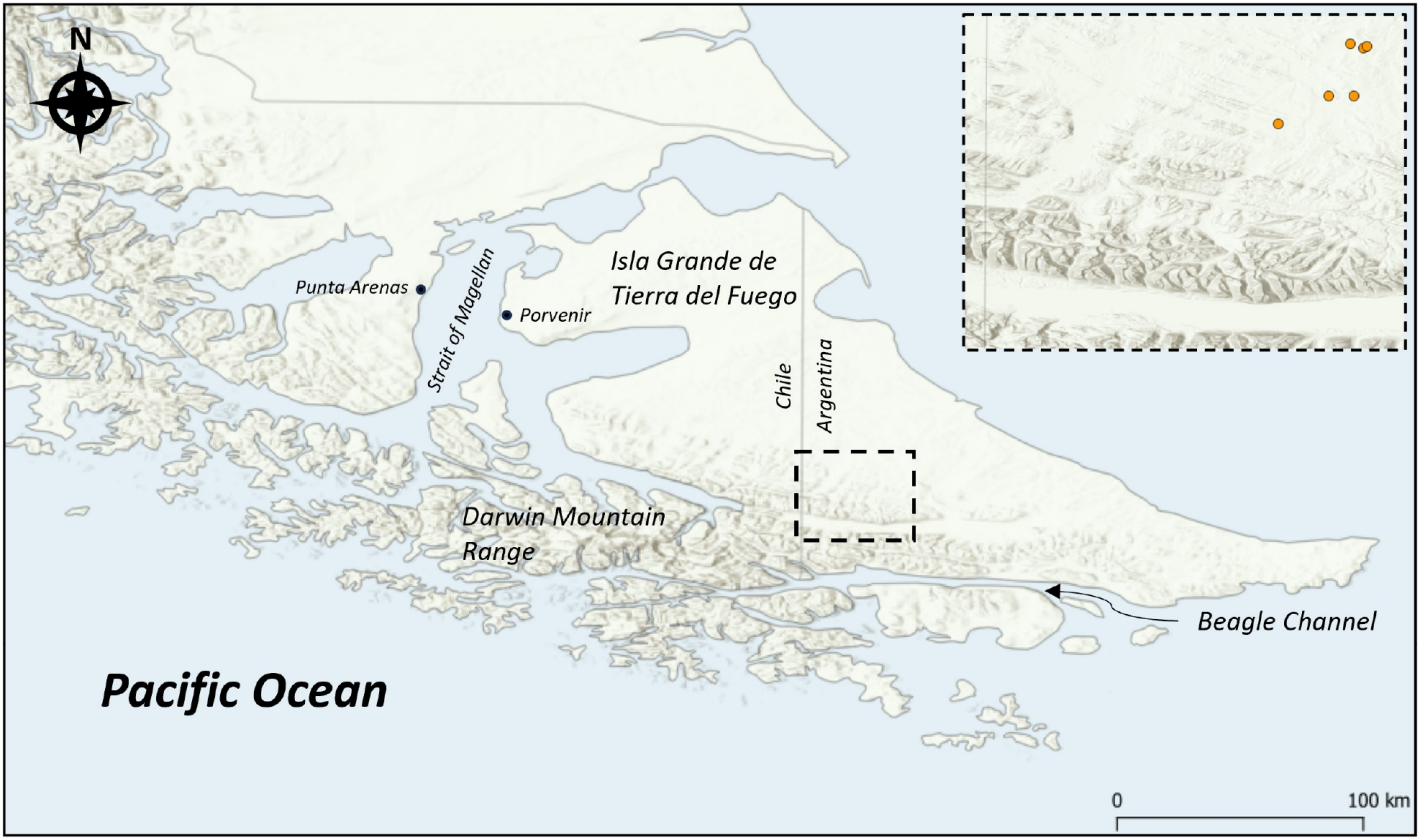
Map of Tierra del Fuego Island (Isla Grande de Tierra del Fuego). The centre line denotes the border between Chile and Argentina. The study area is marked by the dashed rectangle with the camera stations marked inset by orange circles.

Camera traps were set by the Wildlife Conservation Society to monitor feral (free‐living) dogs on Tierra del Fuego Island, Argentina (Schiavini, 2018) (Figure 1). The climate is considered to be subpolar oceanic, with strong winds throughout the short summers and long winters (Zurita et al., 2024). Flat steppes dominated by grass and shrubs are common in the north and central parts of the island, with forests more common in the south parts of the island (Zurita et al., 2024). The island experiences wide shifts in sunlight across the year, typically lacking an astronomical night between November and February, increasing to over 12 hours between June and July.

Camera traps were set at a total of six open‐pasture sites for approximately 3–5 months ($\bar{x}$ = 119.7 ± SD = 28.3 days, range 84–143 days) encompassing March to October. Camera traps were placed in areas to maximize detection of free‐living dogs and wildlife, often on trails along fences (*n* = 4), with the areas along fences baited with fat. The mean distance from each camera trap to the nearest and furthest camera trap was 2.79 km and 15.5 km respectively, covering an area of 30.73 km^2^. Camera traps were placed at approximately knee height and parallel to the ground. All camera trap images were reviewed by a biologist for accuracy (Kays et al., 2022) in Wildlife Insights, and the data used in the analyses are freely available at http://n2t.net/ark:/63614/w12000531.

We used program R version 4.2.3 (R Core Team, 2023) for our statistical analyses. To reduce pseudo‐replication in the dataset, we combined consecutive images of the same species within 30 minutes into the same event (Avrin et al., 2023; Lucherini et al., 2009). We calculated the detection rate (DR) of chillas using the number of independent events for as:

DR=events/trap nights×100.

We assessed whether bait at cameras affected detection rates of chillas by using a *t*‐test.

We applied kernel density estimation methods (Ridout & Linkie, 2009) to quantify the activity patterns of chillas. We transformed the time of each event to radians and then used the *overlap* package (Meredith & Ridout, 2017) in program R to fit the data to a circular kernel density and estimated the activity level at each time period from the distribution of the kernel density based on time of day. We then transformed the time to radians corresponding to sun time in the study area and then repeated the previous analyses.

## 3 RESULTS

Across all cameras, chillas were documented 449 times. Among the six cameras, chillas were documented on average 74.8 times (±49.6 SD, range 17–148). This resulted in an average detection rate of 61.7 (±33.3 SD, range = 13.5–105.7) chillas per 100 trap nights. Detection rates of chillas did not vary significantly based on whether bait was used at the camera trap (*df* = 4, *t* = 0.19, *p* = .85). Free‐living dogs were detected less frequently than chillas (detection rate = 10.7 ± 4.5 SD, range = 0.0–27.7), while culpeo were only detected twice (detection rate = 0.3 ± 0.2 SD, range = 0.0–1.2).

**FIGURE 2:**
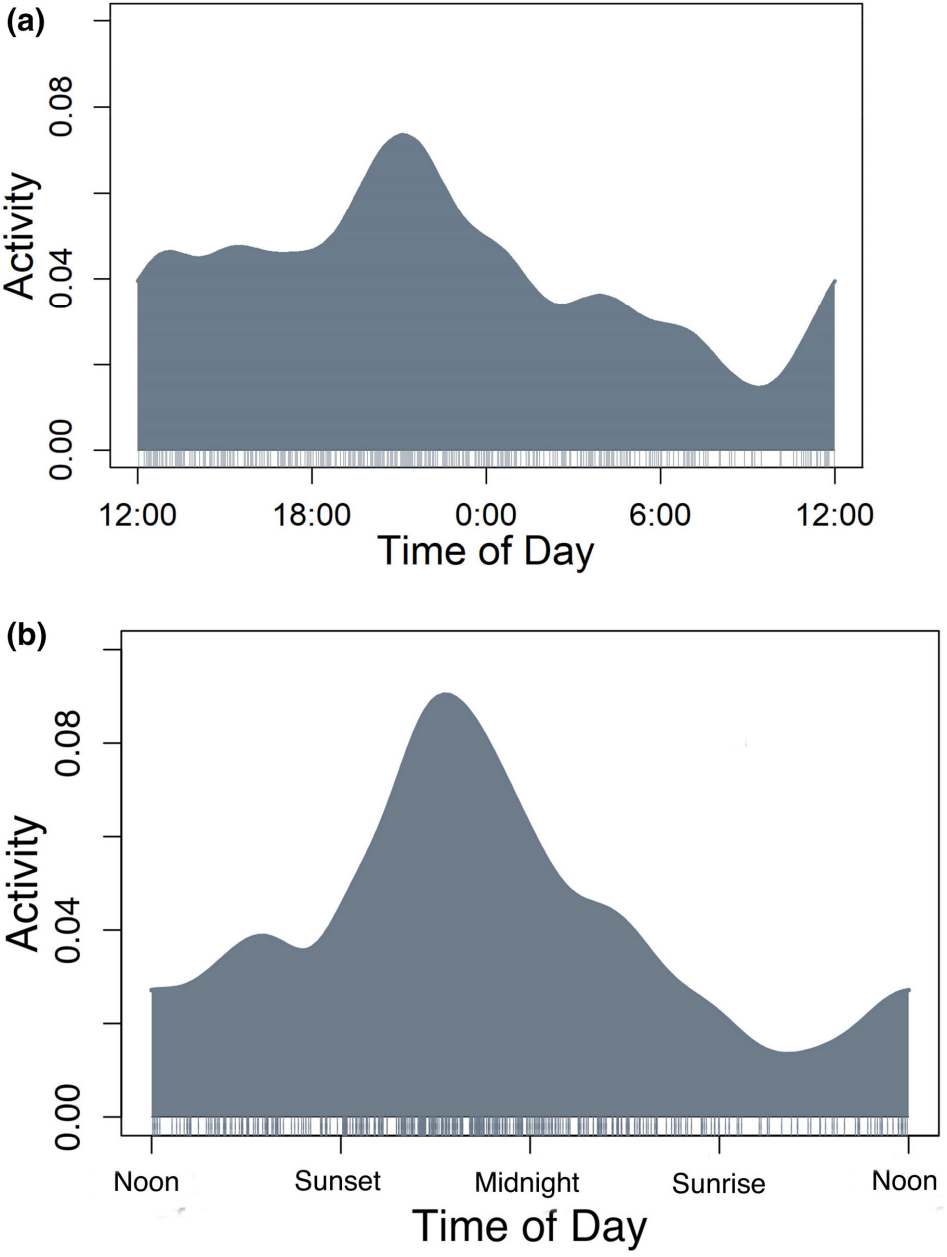
Temporal activity of chillas in Tierra del Fuego by (a) clock time and (b) sun time.

When analysed by time of day, chillas exhibited a cathemeral pattern with increased activity from noon until midnight, with the most activity occurring just before 10 pm (Figure 2a). When analysed by sun time, however, the chillas showed a distinct nocturnal pattern with a clear peak closer to midnight (Figure 2b).

## 4 DISCUSSION

Although recent surveys have indicated a decline of chillas in the Chilean part of Tierra del Fuego Island (Zurita et al., 2024), the relatively high counts across the six cameras in this study underscore their notable continued presence within the Argentinian study area. With an average of around 75 sightings per camera and an estimated average detection rate of 61.7 chillas per 100 trap nights indicate chillas are relatively common in the surveyed habitat. It is important to note, however, that these cameras were not set in a rigorous fashion to estimate chilla abundance and were instead bycatch of a study focused on free‐living dogs. There are also potential limitations associated with camera trapping methodology, including variations in detection probabilities across habitats. The relatively frequent documentation of chillas, however, suggests camera traps placed in a systematic fashion may be a valid method for obtaining rigorous population estimates through n‐mixture modelling or similar methods for informing management strategies, which could be performed across multiple years to understand trends in their populations. Given our survey focused on open‐pasture/steppe habitats, it is important that future work also investigates these trends across the diverse habitats on the island.

The observed differences in activity patterns of chillas when using clock time versus sun time present an intriguing dichotomy and highlight the complexity of accurately estimating temporal activity. Analysis by time of day revealed a cathemeral pattern, but when analysed by sun time, chillas displayed a distinct nocturnal pattern, characterized by a clear pyramidal pattern with a peak around midnight. With the dramatic shifts in daylight in the study area throughout the year, from as little as 7:15 hours of daylight in June–July to 17:15 in December–January, it is important to account for environmental cues when estimating activity patterns. Previous estimates of temporal activity indicated a crepuscular pattern (Zurita et al., 2024), but were only using visual observations from 19:00 to midnight. This may also be indicative of survey type, as camera trapping is effective for documenting cryptic species during all 24 hours across long time periods, while transect surveys are often more effective for documenting diurnal species. Further research exploring the underlying mechanisms driving these temporal activity patterns is warranted to determine if adaptive strategies are employed by chillas, and if these vary in Tierra del Fuego (where they are an invasive species) compared to their native range.

On the mainland of Chile, chilla were found to spend most of their nocturnal active periods in prairies and forest plantations, but used native forest and forest plantations most frequently during their inactive periods, all potential strategies to avoid encounters with free‐living dogs (Silva‐Rodríguez et al., 2010). As all of our sites were from open‐pasture habitats, the nocturnal activity patterns we observed further corroborate that chilla activity patterns may represent avoidance of domestic dogs. Chilla have also been shown to have a moderate dietary overlap (~63%) with the larger culpeo fox (Jaksić et al., 2002). Where the two species co‐occur on the mainland, culpeo appear dominant, exhibiting a strong effect on chilla population dynamics, potentially leading to resource and habitat partitioning (Novaro et al., 2004). Future research should therefore consider placing camera traps across the island and explore temporal‐niche partitioning among these sympatric canids. Systematically expanding the number of camera traps across the various regions and habitats on the island could also help identify where and when interactions between chilla, native fauna and domestic dogs may occur. Several diseases and parasites have been reported across the sympatric foxes (chilla and culpeo) and free‐living dogs on the island (Zurita et al., 2024), with positive cases of Taeniidae (*Leptospira sp*) and scabies in chilla that may be affecting their survivorship (Eisenman et al., 2023; Moya et al., 2019; Verdugo et al., 2016). Chilla are also known to be a host of the protozoan parasite *Sarcocystis guanicoecanis* on Tierra del Fuego Island, but a more complete understanding of their role in the transmission and life‐cycle of this and other diseases and parasites is vital for safeguarding native fauna. Expanding the range and intensity of camera sampling may also reveal insights into chilla prey selection—this information may be critical considering their predatory effects on native fauna are yet to be confirmed (Silva & Saavedra, 2008).

The findings presented in this study provide novel insights into the detection rates and temporal activity patterns of chillas on the Argentinian side of Tierra del Fuego Island as a reference point for future studies. Ecological studies play a pivotal role in unravelling the basic patterns of ecology and behaviour exhibited by species, whether they are a common or invasive species (or both as is the case of chilla in Tierra del Fuego). Detection rates are a potentially limited proxy for true abundance, and management would benefit from rigorous abundance estimates in the future. At the same time, our results stress the importance of using sun time in future studies estimating activity patterns. The insights from this study underscore the importance of employing rigorous methods to inform evidence‐based management strategies aimed at mitigating the impacts of invasive species on native biodiversity and ecosystem integrity.

## AUTHOR CONTRIBUTIONS

**Maximilian L. Allen:** Conceptualization (lead); formal analysis (lead); methodology (equal); writing – original draft (lead); writing – review and editing (equal). **Andrew T. L. Allan:** Conceptualization (supporting); formal analysis (supporting); methodology (supporting); writing – original draft (supporting); writing – review and editing (equal).

## FUNDING INFORMATION

Maximilian Allen received funding for this work from the Illinois Natural History Survey, the Prairie Research Institute and the University of Illinois. Andrew Allan received funding for this work from The Leverhulme Trust (ECF‐2023‐318).

## CONFLICT OF INTEREST STATEMENT

The authors declare they have no conflicts of interest.

## Supporting information


Supplementary Material S1.

Supplementary Material S2.

Supplementary Material S3.

Supplementary Material S4.

## Data Availability

The data used in the analyses are freely available from the Wildlife Conservation Society via Wildlife Insights at: http://n2t.net/ark:/63614/w12000531 and are also included as supplementary material.
